# Supplementary material for: New Weed Hosts for Tomato Brown Rugose Fruit Virus in Wild Mediterranean Vegetation
Source: Plants (Basel). 2022 Sep 1;11(17):2287. doi: 10.3390/plants11172287 (PMC9460064; doi:10.3390/plants11172287)
Supplement: Supplementary file 1 [file plants-11-02287-s001.zip › plants-1863358-supplementary.pdf]

Figure S1

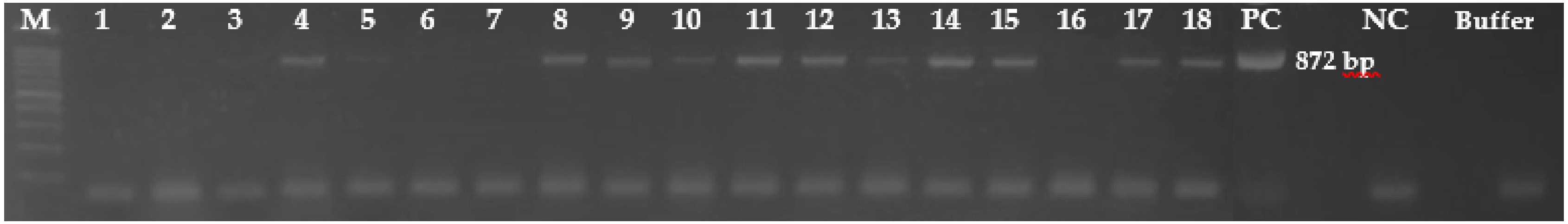

RT-PCR detection of the presence of Tomato brown rugose fruit virus (ToBRFV) in 18 samples of weed plants (lanes 1–18) collected from tomato fields. Lanes 1-18; *Solanum nigrum*, *Malva parviflora*, *Amaranthus retroflexus*, *Chenopodium murale*, *Veronica syriaca*, *S. elaeagnifolium*, *Corchorus olitorius*, *Taraxacum officinale*, *Beta vulgaris*, *S. nigrum*, *B. vulgaris*, *Oxalis corniculata*, *A. retroflexus*, *Conyza canadensis, O. corniculata*, *C. murale*, *M. parviflora*, and *S. elaeagnifolium*, respectively. PC is a positive control (ToBRFV-infected tomato plant). NC is a negative healthy tomato plant control. The specific size of amplicon (872 bp) is shown on the right. Lane M represents a 100 bp DNA ladder (GeneDirex).
